# Enhancing attention in children using an integrated cognitive-physical videogame: A pilot study

**DOI:** 10.1038/s41746-023-00812-z

**Published:** 2023-04-12

**Authors:** J. A. Anguera, M. A. Rowe, J. J. Volponi, M. Elkurdi, B. Jurigova, A. J. Simon, R. Anguera-Singla, C. L. Gallen, A. Gazzaley, E. J. Marco

**Affiliations:** 1 Neuroscape Center, Department of Neurology, University of California, San Francisco, USA; 2 Department of Psychiatry, University of California, San Francisco, USA; 3 Department of Neurodevelopmental Medicine, Cortica Healthcare, San Rafael, USA

**Keywords:** Attention, Cognitive control

## Abstract

Inattention can negatively impact several aspects of a child’s life, including at home and school. Cognitive and physical interventions are two promising non-pharmaceutical approaches used to enhance attention abilities, with combined approaches often being marketed to teachers, therapists, and parents typically without research validation. Here, we assessed the feasibility of incorporating an integrated, cognitive-physical, closed-loop video game (body-brain trainer or ‘BBT’) as an after-school program, and also evaluated if there were attention benefits following its use. Twenty-two children (7–12 years of age) with a range of attention abilities were recruited to participate in this proof of concept, single-arm, longitudinal study (24 sessions over 8 weeks, ~30 min/day). We interrogated attention abilities through a parent survey of their child’s behaviors, in addition to objective performance-based and neural measures of attention. Here we observed 95% compliance as well as, significant improvements on the parent-based reports of inattention and on cognitive tests and neural measures of attention that were comparable in scale to previous work. Exploratory measures of other cognitive control abilities and physical fitness also showed similar improvement, with exploratory evaluation of retained benefits on the primary attention-related outcomes being present 1-year later. Lastly, there was no correlation between the baseline parent-rated inattention score and the improvement on the primary task-based measures of attention, suggesting that intervention-based benefits were not solely attained by those who stood the most to gain. These pilot findings warrant future research to replicate and extend these findings.

## Introduction

Children fall along a spectrum when it comes to their attention abilities, with issues of inattention differentially impacting distinct aspects of their life such as their home, school, and community function^1^. On one end of the spectrum, there are children who do not demonstrate distractibility, impulsivity or hyperactivity that interfere with function at home or school but who will show variability in cognitive control metrics and neural signatures of attention when tested in typical laboratory settings. On the other end of this spectrum are children meeting a clinical diagnosis of Attention Deficit and Hyperactivity Disorder (ADHD), who typically show heightened intra-individual variability beyond typically developing children on such objective measures. For this reason, it is critical, to consider interventions with a vantage point that spans across children with a range of abilities, rather than those approaches that are assumed to be ‘one size fits all’.

The most common and research tested effective intervention for ADHD, stimulant medication, can lead to headaches^2^, insomnia^3–5^, nausea^6,7^, emotional outbursts^8,9^, and in some children, psychosis^10,11^. Clearly, a non-pharmaceutical treatment that improves attention for children with ADHD generally, or specific cognitive control challenges more precisely, would be a welcome alternative (or augmentation to the current pharmacologic options), as well as for “neurotypical” children who may also benefit from a precision medicine approach. Indeed, there is a growing appetite for such advances, as evidenced by a recent FDA-cleared non-drug treatment for inattention in ADHD^12^. Even beyond this example, it is clear that parents are committed to finding options to help their children, both with and without ADHD, to enhance their attention abilities.

The two most prevalent non-drug intervention strategies explored to enhance attention in children have involved cognitive training^13–16^ and physical exercise^17–22^. Recent work by our own group and others have demonstrated the benefits of targeted attention interventions in children with issues of inattention^16,23–25^. Similarly, fitness-based interventions have been shown to improve cognitive control abilities (defined here as attention, working memory, and cognitive flexibility^26,27^) in typically developing children^28–31^, including those with issues of inattention^32^. Such approaches aimed at enhancing cognitive control abilities are especially intriguing, given a number of studies that have demonstrated strong links between cognitive control abilities and a wide range of real-world outcomes for children including academic performance, literacy, health, and well-being^33–35^. Combined cognitive and physical interventions, typically presented in the form of ‘exergames’, is an especially compelling approach to benefit both cognitive and physical health, while also being time- and resource-effective. Indeed, one exergaming study involving children with ADHD demonstrated behavioral benefits on laboratory measures of executive function following the intervention beyond that of a wait-list control group^36^. However, these studies (and other exergaming studies with typically developing children^37,38^) have not explored how improvements on such metrics align with parental perceptions of inattention, the underlying neural mechanisms of these improvements, or how long the benefits last.

**Fig. 1:**
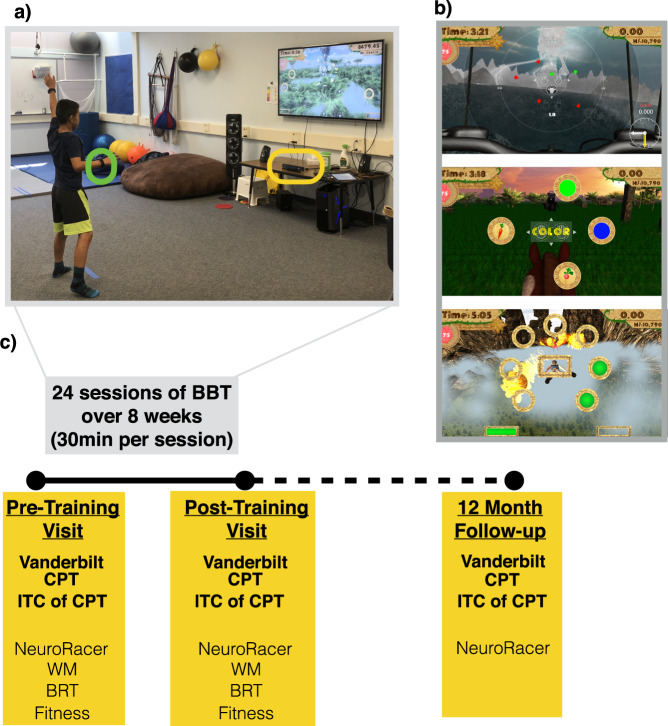
BBT platform. **a** Image of participant playing BBT. Highlighted is the use of a heart rate monitor (green circle; used to assess and adapt the physical intensity of game play in real-time) and the Microsoft Kinect™ motion capture technology (yellow circle; used to collect responses with one’s hands and/or feet based on the cognitive task presented on the monitor and adapt the cognitive difficulty of each game in real-time). **b** Image of the three modules in BBT, with the top panel showing the visual search module, the middle panel showing the task switch module, and the lower panel showing the working memory module. A video of BBT in action can be viewed at https://youtu.be/vvR5WhSzQU4. **c** An overview of the study timeline with outcome measures collected at each timepoint listed. Abbreviations - CPT: Continuous Performance Task. ITC of CPT: Inter-Trial Coherence of the Continuous Performance Task. WM: Working Memory Task. BRT: Basic Response Time Task.

A critical question is whether an integrated cognitive and physical training intervention is a practical approach to deliver outside of the laboratory setting. To assess this question, the present study was not conducted in a traditional lab, but instead at a local elementary school as an after-school program. Second, we chose an ‘all-comers’ approach for participation to examine how beneficial this intervention is for children across a spectrum of attention abilities. This allows for the exploration of whether the training confers benefits to children with a broad swath of abilities or only to those with the greatest, “clinically significant” impairments. Third, it is important to demonstrate evidence of an intervention’s impact on cognitive and neural laboratory tests of attention, as well as real-world function as assessed by parents. Finally, should such an approach show positive results, a critical question is if any of the realized benefits persist beyond the initial treatment period. To address these questions, we conducted a single-arm, open label, proof of concept pilot study (including a 1-year follow-up) of our combined cognitive/physical intervention, body brain trainer (BBT^39^; see Fig. 1). We also compared the observed improvements on our primary outcome measures to analogous assessments in our previous work^16^ for both typically developing children, as well as those with ADHD.

## Results

### Demographics and feasibility metrics

**Fig. 2:**
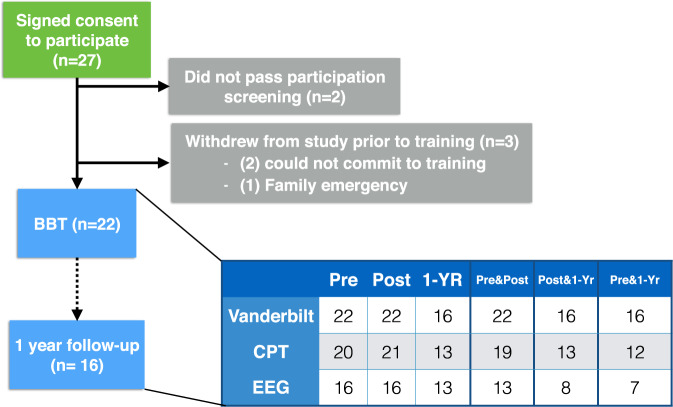
CONSORT diagram. Illustration depicting the number of participants at different stages of the study, from consent to enrollment and 1-year follow-up. The embedded table reflects the number of participants with datasets for each of the primary measures of interest at each time point, as well as the number of individuals with data at each paired timepoint. For the CPT task, missing data was due to participants not being able to stay to the end of their testing session due to other outside obligations those days. For the EEG recordings, missing data points reflect a hardware error where photodiodes used to time lock the onset of targets were not functioning properly, preventing the analysis of event-related activity.

Twenty-seven children, attending Neil Cummins Elementary School in Corte Madera California, between the ages of 7 and 12 years of age were recruited for participation through school postings, newsletters, and word of mouth describing a study for children designed to enhance attention (see Supplementary Figure 1). Of the 27 individuals screened for enrollment, two failed our eligibility screening and three dropped out after enrolling, but prior to beginning training (see CONSORT diagram, Fig. 2). Thus, a total of 22 children (6 female) ages 7–12 years (mean age = 9.24, SD = 1.57) completed the intervention, with 16 contributing data to the 1-year follow-up assessment (see Supplementary Table 1 for demographic and WISC-V information). The number of individuals with primary data outcomes collected at each time point as well as an overview of the reasons for missing data are described in Fig. 2.

On average, participants completed 95.8% of the required 24 sessions (mean = 23.0 ± 2.5 sessions). During the BBT intervention itself, participants demonstrated improved accuracy (t_(21)_ = −7.3, *p* < 0.001) and increased heart rate (t_(21)_ = −3.5, *p* = 0.002) when comparing initial training sessions (the average performance over the 1st two days of training) versus later training sessions (the average performance over the final two days of training), after collapsing across modules and levels (see Supplementary Figure 2), suggesting that participants improved on cognitive measures and showed increased physical effort across the training period on the BBT intervention.

### Primary outcome measures

**Fig. 3:**
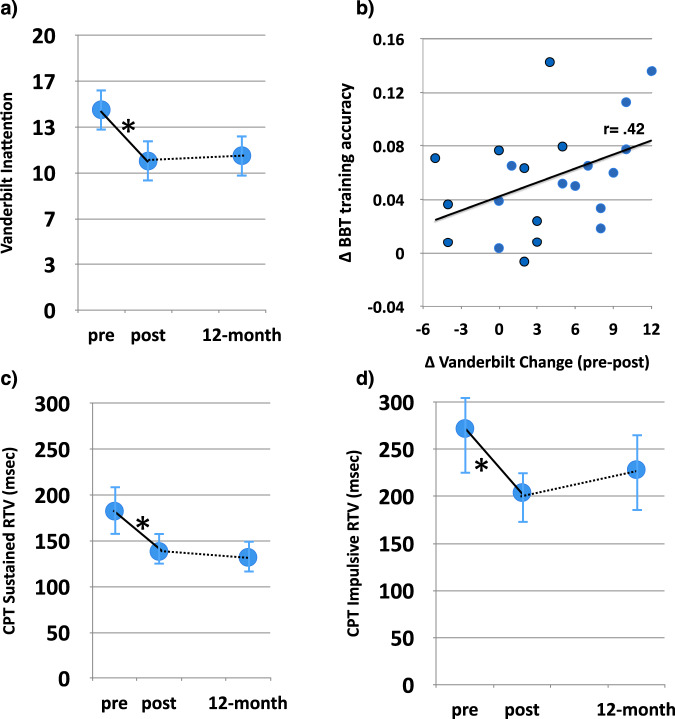
Parent report of inattention and CPT task over time. **a** Parent report of inattention (Vanderbilt) over time illustrating the group mean at each time point. **b** Scatterplot illustrating trending correlation between the change on the Vanderbilt inattention measures versus overall change in accuracy collapsed across BBT training modules (*r* = 0.42, *p* = 0.05). **c** CPT (sustained condition) response time variability over time illustrating the group mean at each time point. **d** CPT (impulsive condition) response time variability over time illustrating the group mean at each time point. *p < 0.05. Error bars represent s.e.m.

To test whether the intervention led to changes in parent perceptions of inattention^16^, we examined change on the Vanderbilt parent report measure at baseline and following the completion of the intervention based on a cumulative total from questions 1–9 on the questionnaire. We observed a significant decrease in parent observed inattentive behaviors (average change = 3.72 points, t_(21)_ = 3.65, *p* = 0.002, Fig. 3a; see Table 1 for group values and effect sizes). Furthermore, 16/22 showed a positive improvement in their score following training, with 7 out of the 8 of individuals who initially met the Vanderbilt research criteria for ADHD no longer meeting criteria after training. Additionally, we observed a positive correlation between the change in Vanderbilt (pre-post) and average change in training accuracy on BBT (r_(22)_ = 0.42, *p* = 0.05; Fig. 3b), revealing that those participants who demonstrated the greatest performance improvements on the BBT training showed the greatest gains in parent-reported inattention. With respect to assessing the stability of these changes 1-year later, the inattention score at the 1-year follow-up was nearly identical to that at post (*t*_(15)_ = 0.001, *p* = 0.99), suggesting these improvements persisted 1 year after training. However, this interpretation should be taken with caution given that performance at the 1-year mark was not significantly different than that observed at baseline (t_(15)_ = 1.57, *p* = 0.14).

**Table 1:** Primary outcome measures.

		Pre-Training Mean (SD)	Post-Training Mean (SD)	Change Score Mean (SD)	Cohen’s d	1-Year Mean (SD)
Parent Report	Vanderbilt Inattention	14.59 (5.29)	10.86 (3.34)***	+3.73 (4.79)	0.78	11.25 (5.46)†
CPT	Sustained RTV	184.77 (71.74)	141.57 (41.21)*	-43.20 (76.4)	0.57	132.03 (50.21)†
	Impulsive RTV	268.04 (119.49)	183.97 (79.47)***	-84.06 (95.79)	0.88	228.37 (139.20)†
Neural	mfTheta ITC Sustained	0.09 (0.04)	0.14 (0.08)*	+0.052 (0.084)	0.61	0.19 (0.12)†
	mfTheta ITC Impulsive	0.10 (0.08)	0.15 (0.06)*	+0.051 (0.072)	0.71	0.18 (0.07)†

^*^p < 0.05 compared to pre-training mean.

^**^p < 0.01 compared to pre-training mean.

^***^p < 0.005 compared to pre-training mean.

^†^*p*-value not significant at p < 0.05, compared to post-training mean.

Cohen’s d: Reflects the effect size comparison of pre to post-training.

Our measure of sustained attention was a modified version of a well-validated continuous performance task (CPT), the Test of Variables of Attention (TOVA)^40,41^, which provides an index of sustained attention and impulsivity and has been used as an outcome measure in previous intervention studies^39,42–45^. We observed an improvement on the primary measure of RTV for both the sustained (t_(18)_ = 2.46, *p* = 0.024) and impulsive conditions (t_(18)_ = 3.83, *p* = 0.001; see Fig. 3c, d). Similar to the Vanderbilt findings, no differences were present between performance at post and at the 1-year mark for either condition (t_(12)_ < 1.01, *p* > 0.34). However, there was a difference at the 1-year mark compared to baseline for the sustained condition (t_(11)_ = 2.68, *p* = 0.02), but not for the impulsive condition (t_(11)_ = 0.70, *p* = 0.50), highlighting the sustained impact BBT had on this particular condition of the CPT task. The means and standard deviations are reported in Table 1 along with the values on all other commonly reported variables from this task in Table 2.

**Table 2:** Secondary and Exploratory Outcome Measures.

		Pre-Training Mean (SD)	Post-Training Mean (SD)	Cohen’s d	1-Year Mean (SD)
CPT	Sustained RT	491.33 (89.77)	459.80 (88.91)*	0.53	451.00 (103.45)†
	Impulsive RT	453.37 (128.02)	390.35 (95.94)*	0.64	398.98 (111.56)†
	Sustained dPrime	2.71 (1.17)	4.38 (1.67)***	−1.28	4.49 (1.54)†
	Impulsive dPrime	1.70 (0.67)	2.37 (0.91)***	−0.89	1.95 (2.02)†
	Sustained Tau	159.20 (80.95)	134.29 (53.96)	0.26	108.94 (54.79)†
	Impulsive Tau	231.72 (133.81)	147.85 (80.04)**	0.74	170.98 (107.33)†
NeuroRacer	Driving Accuracy	48.54 (22.10)	64.00 (22.68)***	−0.89	65.72 (15.56)†
	Multitasking dPrime Ratio	−0.53 (0.34)	0.05 (0.59)***	−0.93	0.02 (1.00)†
AID	Accuracy	0.63 (0.13)	0.69 (0.13)	−0.47	--
	RT	754.51 (184.57)	790.18 (213.82)	−0.19	--
BRT	RT	357.68 (64.55)	338.90 (49.60)	0.31	373.87 (156.72)†
	RTV	101.26 (26.16)	110.59 (24.55)	−0.25	357.57 (171.66)
Physical Outcome Measures	Curl Up	20.35 (18.84)	28.80 (24.14)*	−0.53	--
	Max Heart Rate	187.50 (10.60)	174.50 (22.97)*	0.56	--
	Trunk Lift	6.25 (2.43)	7.88 (2.49)	−0.44	--
	Push Up	5.75 (6.87)	7.05 (8.00)	−0.24	--
	Pacer	17.60 (10.66)	18.50 (9.90)	−0.16	--
	Max V02	41.14 (2.83)	41.91 (2.76)	−0.21	--
IQ	WISC-V PSI	96.20 (7.11)	--	--	105.20 (9.65)*

^*^p < 0.05 compared to pre-training mean.

^**^p < 0.01 compared to pre-training mean.

^***^p < 0.005 compared to pre-training mean.

^†^*p*-value not significant at *p* < 0.05, compared to post-training mean.

**Fig. 4:**
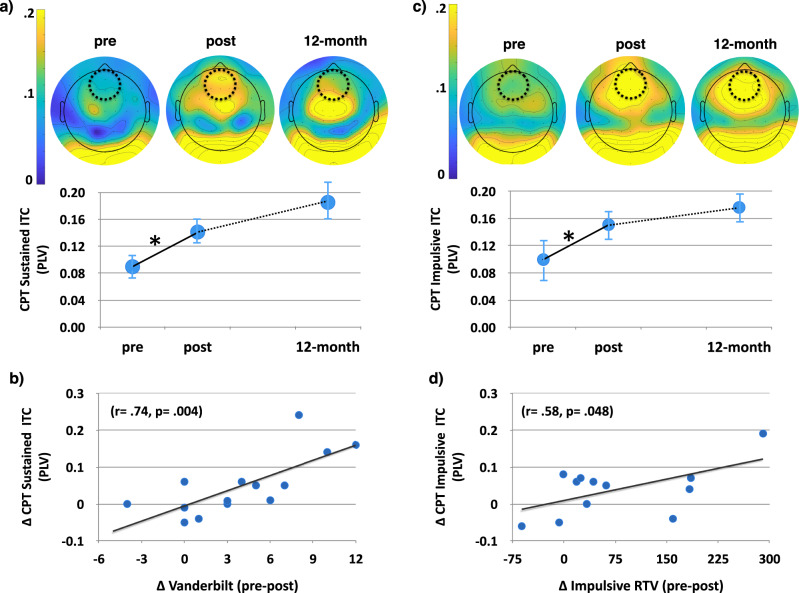
Neural correlates of each CPT task over time. **a** Midline frontal theta inter-trial coherence (ITC) for the sustained condition of the CPT over time. **b** Correlation between the change in sustained ITC and the change in Vanderbilt inattention measure. **c** Midline frontal ITC for the impulsive condition of the CPT over time. **d** Correlation between the change in impulsive ITC and the change in impulsive RTV. The dashed circle on the topographic plot illustrates the electrodes where statistical analyses took place. *p < 0.05. Error bars represent s.e.m.

While participants performed the CPT task, EEG activity was recorded with Active Two head cap (Cortech-Solutions) with a BioSemi ActiveTwo 64-channel EEG acquisition system in conjunction with BioSemi ActiView software (Cortech-Solutions). Based on our previous work^42^, we chose to examine inter-trial coherence (ITC) for the neural correlates associated with performance during each condition of the CPT task. ITC assesses the electrophysiological response consistency of activity at a given region and reflects the extent to which synchronization occurs from trial to trial in EEG at a particular frequency and latency. ITC has been shown to be correlated with RTV^42^ and has been shown to be sensitive to intervention-based changes. ITC has been implicated in sustained attention abilities^43,46,47^, including correlating with RTV across the lifespan^48^. For the sustained condition, we observed a session by time window interaction (F_(11,132)_ = 2.45, *p* = 0.04), suggesting that there was a differential increase over session at distinct time-windows (Fig. 4a). To mitigate issues of multiple comparisons, we narrowed our analyses to the time window that had the greatest, or ‘peak’, activity when collapsed across session, as in our previous work^43,47,49^. Follow-up tests at the time-window of peak ITC, 100–150 m, revealed a significant increase in ITC following the intervention (t_(12)_ = 2.20, *p* = 0.05). The change in ITC at this time window also showed a correlation with the observed improvement on the Vanderbilt parent report (r_(13)_ = 0.74, *p* = 0.004, Fig. 4b), that survives an FDR correction for multiple comparisons (*p* = 0.04).

For the impulsive condition, we observed a main effect of session (F_(1,13)_ = 7.12, *p* = 0.02), a main effect of time-window (F_(11,143)_ = 14.74, *p* = 0.0001), but no session by time-window interaction (F_(11,143)_ = 1.16, *p* = 0.32), suggesting that while there was an increase in ITC during this condition following the intervention, it was not specific to any particular time-window unlike the sustained condition (Fig. 4c). The change in ITC collapsed across all time windows was correlated with the observed RTV improvement on the impulsive CPT condition (r_(12)_ = 0.58, *p* = 0.05, Fig. 4d), unlike the sustained condition (r_(11)_ = 0.23, *p* = 0.49; correlations across all primary measures are displayed in Supplementary Table 2).

No differences were present when comparing post-training performance to that at the 1-year mark for the sustained condition (Z_(8)_ = 0.98, *p* = 0.33), as well as for the impulsive condition (collapsing over all time windows, Z_(8)_ = 1.12, *p* = 0.26), although similar null findings were also present for comparisons between baseline and the 1-year time point in each case (sustained: Z_(7)_ = 0.86, *p* = 0.40; impulsive: Z_(7)_ = 1.40, *p* = 0.16). All possible correlations across the primary metrics of interest are shown in Supplementary Table 2.

### Correlations between baseline Vanderbilt score and change in primary outcomes

Given that our recruited population was heterogenous with respect to attention issues, we examined whether there was a relationship between the extent of inattention at baseline, as measured by the score on the Vanderbilt, with the change in performance following the intervention on each of our task-based primary outcome measures. This correlational analysis revealed no significant relationship between baseline Vanderbilt inattention score and the extent of improvement on any of our primary measures of interest: CPT Sustained RTV (r_(19)_ = 0.35, *p* = 0.14); CPT Impulsive RTV (r_(19)_ = 0.12, *p* = 0.63); ITC Sustained (r_(13)_ = 0.40, *p* = 0.17); ITC Impulsive (r_(14)_ = 0.08, *p* = 0.77).

### Secondary outcome measures

The first secondary outcome measure was the NeuroRacer multitasking assessment, a measure used in our previous work^43,47,50^ that compares performance during a perceptual discrimination task under dual- vs. single-tasking conditions. The cognitive multitasking metric revealed an improvement over time (t_(19)_ = 4.17, *p* = 0.001, see Table 2), suggesting that there was an improvement in multitasking performance following training. Performance at the 1-year mark showed no difference versus performance at the post-training mark (t_(13)_ = 0.19, *p* = 0.85), although similar null findings were also present for comparisons between baseline and the 1-year time point (t_(10)_ = 1.162, *p* = 0.14). Similar findings were present for visuomotor tracking, with improvement observed following training (t_(19)_ = 4.10, *p* = 0.001) and at the 1-year mark versus baseline (t_(11)_ = 4.63, *p* = 0.001), with no changes at the 1-year mark when compared to post-training (t_(13)_ = 0.45, *p* = 0.66).

Another of our secondary outcome measures was a delayed recognition working memory task used in previous intervention studies^43,51^ to measure changes in participants’ ability to maintain an accurate mental representation of items in working memory either in presence or absence of distracting or interfering information. Here we observed a trend towards improvement in performance accuracy (t_(16)_ = 1.95, *p* = 0.07), see Table 2), with no improvement on RT during this task (t_(16)_ = 0.80, *p* = 0.44).

The physical outcome measures (the remaining secondary outcome measures) were performed before and after training, and include elements from the FitnessGram^52–58^, a field-test battery for youths used by the Presidential Youth Fitness Program that has established standards for ages 5–17 years. Participants performed a Curl-Up, 90 degree Push Up, Trunk Lift, and the PACER run to assess changes in fitness and strength. We observed improvements following training on the Curl-Up (t_(19)_ = 2.35, *p* = 0.03) and max HR (t_(19)_ = 2.50, *p* = 0.02), with trends towards improvements on the Trunk Lift (t_(19)_ = 1.95, *p* = 0.06, see Table 2). No change was observed on the Push Up (t_(19)_ = 1.06, *p* = 0.30), PACER number of laps completed (t_(19)_ = 0.72, *p* = 0.47), or VO_2_max (t_(19)_ = 0.94, *p* = 0.36) measures following training.

Finally, we administered a measure of basic response time to ensure that any differences we see are not due to differences in motoric quickness. Thus, this task acts as a control measure, where we would expect no changes in performance, compared to other outcomes where we hypothesize there will be significant improvements over time. Here we observed no change on RT (t_(20)_ = 1.44, *p* = 0.17) or RTV (t_(20)_ = 1.14, *p* = 0.27, see Table 2) following the intervention, suggesting that participants did not improve their basic motoric abilities after BBT and that any RT-based changes on other outcome measures could not be solely attributed to such changes as well.

### Comparison of results to those from other work

**Fig. 5:**
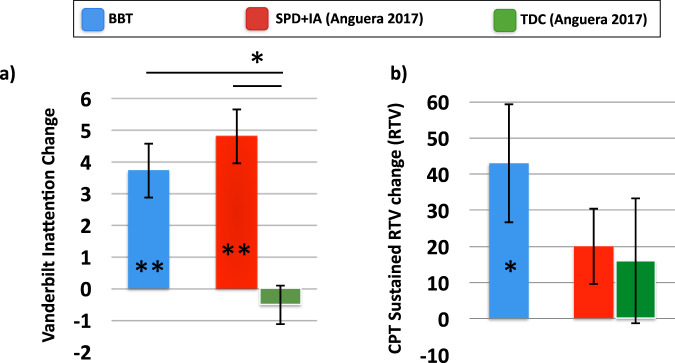
Comparison of primary outcomes to Anguera et al. (2017). **a** Change on Vanderbilt Inattention Measure for BBT cohort and participants from Anguera et al. (2017). **b** Change on CPT task on the Sustained Condition for BBT cohort and participants from Anguera et al. (2017). SPD + IA = Children with Sensory Processing Disorder and ADHD comorbidity. TDC Typically Developing Children. Error bars represent s.e.m. *p < 0.05, **p < 0.01.

Given the single-arm study design and the ‘all comers’ approach for eligibility regarding inattention symptoms, we looked at prior research to provide context regarding the observed improvements, specifically on the primary cognitive metrics. In Anguera et al. (2017)^13^, we reported how typically developing children (TDC), as well as children with diagnosed issues of both inattention and sensory processing disorder (SPD_IA_), responded on the Vanderbilt and TOVA following the use of a different digital therapeutic (Project: EVO, or AKL-T01). In that study, those children with inattention issues (SPD_IA_) improved on the inattention portion of the Vanderbilt (mean change = + 4.81; see Fig. 5 and Table 3 for values) to a similar extent as the children in the present cohort (mean change = + 3.73), whereas no such improvement was observed in the typically developing children as expected (mean change = −0.51). Using a repeated measures ANOVA analysis with a factor of session (pre, post) and between-group factor (BBT, TDC, SPD_IA_), we observed a main effect of session (F_(1,55)_ = 29.60, *p* ≦ 0.0001) as well as group by session interaction (F_(2,55)_ = 5.04, *p* = 0.01), suggesting that there was a differential improvement amongst these groups over time. Follow-up analyses revealed no group by session interaction between the BBT and the SPD_IA_ groups (F_(1,34)_ = 0.022, *p* = 0.88), suggesting comparable improvement over time both groups. Furthermore, each of these groups showed a significantly larger improvement on the Vanderbilt when directly compared to the TDC group (F_(1,40)_ ≧ 7.67, *p* ≦ 0.008 in each case), as one would expect given that the TDC group had no issues of inattention and minimal improvements on Vanderbilt.

**Table 3:** Comparison of Primary Outcomes to Anguera et al. (2017).

		Pre-Training Mean (SD)	Post-Training Mean (SD)	Outcome Improvement
BBT	Vanderbilt Inattention	14.59 (5.29)	10.86 (3.34)***	+ 3.72^†^
SPD + ADHD (Anguera et al. 2017)	Vanderbilt Inattention	21.43 (3.28)	16.62 (3.50)***	+ 4.81^‡^
TDC (Anguera et al. 2017)	Vanderbilt Inattention	5.24 (3.46)	5.75 (2.69)	− 0.51
BBT	Sustained RTV	184.77 (71.74)	141.57 (41.21)*	+ 43.20
SPD + ADHD (Anguera et al. 2017)	Sustained RTV	189.39 (50.16)	169.41 (41.65)	+ 19.98
TDC (Anguera et al. 2017)	Sustained RTV	137.02 (47.00)	121.01 (41.87)	+ 16.01

*SPD* *+* *ADHD* Children with Sensory Processing Disorder and ADHD comorbidity

*TDC* Typically Developing Children.

^*^p < 0.05 compared to pre-training mean.

^**^p < 0.01 compared to pre-training mean.

^***^p < 0.005 compared to pre-training mean.

^†^BBT improvement was significantly different than TDC (*p* < 0.05).

^‡^SPD + ADHD improvement was significantly different than TDC (*p* < 0.05).

With respect to the CPT task (sustained condition) and RTV metric (see Fig. 5 and Table 3 for values), the same ANOVA approach described above revealed a main effect of session (F_(1,49)_ = 8.43, *p* = 0.006), but no group by session interaction (F_(2,49)_ = 1.04, *p* = 0.36), suggesting that all groups improved equivalently on this metric after training. However, it should be noted that only the BBT group demonstrated a significant improvement following training, unlike the SPD_IA_ and TDC groups in Anguera et al. (2017).

## Discussion

The present findings provide preliminary evidence of positive benefits in cognitive and neural markers of attention across a heterogenous population of children using a novel, integrated, cognitive-physical videogame: BBT. Notably, our primary outcome analyses demonstrated that BBT enhanced performance on a well-established parent-based assessment of inattention following an 8 week intervention, which was comparable in scale to previous work^16,59^. Furthermore, we also evidenced benefits on attention with both cognitive and neural assessments, with these improvements persisting for 12 months. Finally, our exploratory analyses demonstrated that BBT improved performance on other measures of cognitive control, as well as physical fitness. Here, we discuss the practical consideration of this study, as well as the possible mechanisms of these intervention-related enhancements.

A major goal of this study was to determine feasibility of approach. Here we succeeded in delivering the BBT intervention outside of the laboratory as an afterschool activity, comparable to other at-school intervention programs that have reported beneficial effects^60–63^. In these studies, students who played off-the-shelf exergames showed positive effects on physical activity that was comparable, if not greater, than traditional physical education programs. Equally encouraging was the compliance rate observed across a diverse cohort of BBT participants, suggesting that this custom-developed, intervention utilizing closed-loop adaptive mechanics was able to maintain compliance amongst these children in a meaningful fashion. While it cannot be ruled out entirely, we believe that the renumeration given to participating families was not the driving force for the high compliance rates observed, as the rates of attrition was comparable to several other studies using digital interventions. However, there are several practical considerations to consider with respect to replicating these efforts. The required elements for BBT were a dedicated space that could accommodate a large screen/monitor, a desktop Windows PC computer ($529), a Kinect 2™ motion capture camera ($89), an Apple Watch to monitor heart rate monitor ($399, although in other work we have captured these data with a more inexpensive wearable HR monitor (Garmin™, $42), and an individual to oversee the training protocols (here, a research associate). While the space and peripheral equipment needs are not trivial, the possibility of scaling this design in other locations appears to be feasible given that the costs of these technologies are declining^64^.

Here we observed that BBT improves both objective and subjective measures of attention in children with heterogenous attention difficulties, similar to other tablet-based interventions (Akili’s AKL-T01 or a custom meditation application called MediTrain) utilizing closed-loop adaptivity mechanics^17,19^. While this is admittedly a small pilot study, it is still notable that 7/8 of individuals who met the criteria for ADHD using the Vanderbilt no longer met criteria after training. These parent-based reports of improvements also showed persistent, long-lasting effects, which is consistent with recent work involving AKL-T01 that reported comparable parent report benefits persisting 3 years following the intervention in children with issues of inattention^65^. What is unique about the present findings is the nature of the benefits across individuals: that is, there was no correlation between baseline inattention score and improvement on the primary task-based measures of attention. Thus, intervention-based benefits were present across a range of participants, not just those at baseline who stood the most to gain. This is distinct from other digital therapeutic studies where those populations with the greatest impairments have typically been the one’s to show the largest improvements^66,67^. It should be noted that the decision to allow participants without diagnosed ADHD to participate would theoretically predict lesser group benefits on these parent reports, given that BBT sample was quite heterogenous with respect to issues of inattention. Thus, it was surprising, but encouraging, that the present findings demonstrated significant improvements, suggesting the possibility that a homogeneous affected ADHD cohort with BBT might lead to even larger benefits regarding parent reports of improvements in inattention.

Similar to the Vanderbilt findings, the pattern of results for the CPT assessment shows striking similarities to other intervention studies (with control groups) involving children with issues of inattention and/or ADHD^23,49^. In those studies, which utilized the AKL-T01 tablet-based intervention, the improvements observed involved the same metric of RTV examined here, suggesting that BBT improved objective indices of attentional control comparable to these other works. Unlike the aforementioned studies, we also observed persistent performance improvements on the sustained condition 1-year later, illustrating one of the long-lasting potential benefits of the BBT intervention. However, it should be noted that none of the previously referenced works interrogated these metrics during the impulsive condition. Here we observed comparable improvements on each condition, suggesting that the BBT intervention may have the breadth to generalize to different facets underlying attentional abilities.

Here we demonstrated that BBT led to increased midline frontal theta ITC during our CPT task, suggesting that the intervention led to a greater ability to engage attention-based neural control functions that support attention-centric performance. The enhancement of midline frontal theta metrics following similar closed-loop adaptive interventions has been evidenced in other populations (e.g. older adults) as well^43,47,68^, supporting the idea that this neural signature underlies observed cognitive improvements. These findings also extend the idea that digital therapeutics designed to engage specific brain networks can enhance attention-related performance^69–71^. The change in ITC for each condition on the CPT task also showed selective correlations: the improvement on the parent report correlated with ITC during the sustained condition, while RTV on the CPT task correlated with ITC during the same condition. While intriguing, given that such neural-behavioral relationships are useful in informing future assessment and treatments, these exploratory findings must be taken with caution given that this is a pilot study and issues of multiple comparisons have not been accounted for. However, unlike our previous work^16^, we also demonstrate that the BBT intervention led to persistent neural enhancements on each CPT condition 1-year later (albeit in the subsample that returned for this testing). We have previously observed long-term persistence involving midline frontal theta power in older adults six years later following their participation with a closed-loop adaptive intervention (NeuroRacer)^47^. In conjunction with those results, the present findings suggest that digital therapeutics like BBT may have the potential for both immediate and long-term positive persisting outcomes on neural measures.

We also observed improvements on secondary measures of cognitive control and physical fitness following the BBT intervention. The BBT participants reached a near zero cost on the NeuroRacer multitasking assessment, suggesting that their perceptual discrimination abilities improved to the point where they performed comparably when they were ‘single-tasking’ versus ‘multitasking’. As a point of context, this assessment was previously used to evidence multitasking abilities in older adults following a laptop-based cognitive intervention^43^, with those individuals reaching a 13% multitasking cost after training. In that same study, we observed that the older adults improved their working memory abilities on the same delayed recognition task used here. While we only observed a trend suggestive of similar improvements on this task, it is notable that this measure of far transfer showed potential signs of improvement in a very different heterogenous sample involving a closed-loop adaptive intervention. Taken as a whole, the improvements on the CPT task with these other measures assessing cognitive control abilities supports the underlying idea of the BBT platform engaging each cognitive control pillar (attention, working memory, cognitive flexibility) as envisioned in the training prescription. Note that the examination of RT and RTV on the BRT control task revealed no change on either metric over time, supporting the assertion that the cognitive improvements observed were not simply a function of improved speed of processing.

The improvements on the physical fitness metrics collected suggest that the fitness component of the BBT intervention indeed led to a meaningful engagement of the cardiovascular system in these children. It is well known that exercise can lead to improvements in general health for children^29,72–74^, and have a number of positive effects in children with issues of inattention^18,19,75^. Indeed, the link between cardiorespiratory fitness and cognitive control is well-established^76,77^, with cognitive control having been described as an ‘explanatory mechanism’ for the relationship between children’s physical activity and real-world functions such as academic achievement^78^. Similarly, physical fitness has also been shown to play a key role in children’s mental health^79–83^, with greater fitness being associated with improved resilience and decreased anxiety^84,85^. Here we hypothesize that the adaptive physical (and cognitive) components of the BBT intervention enhanced both cognitive control abilities as well as emotional and behavioral regulation through the targeted engagement of prefrontal regions.

The ability to couple two distinct types of training into a singular experience is also an important practical consideration. This is especially pertinent with respect to engaging this particular population in a meaningful way on more than one intervention in the setting of a limited amount of time, bandwidth, and interest. It also provides a secondary option for parents and their children with respect to engaging with these types of treatments, as one could easily imagine individual preferences for “move and play” options versus “sit and play.”

This study represents a first step in understanding the potential benefits of this combined cognitive-physical video game for children across a spectrum of attention abilities. However, there were several limitations in the present work that should be addressed in future studies. First, the single-arm design utilized here only allowed for comparisons to previously collected data sets. While our primary outcome comparisons to historical data provide some context for enhancements observed, this study design cannot directly account of issues of expectation or practice effects. The next approach for understanding improvements would be a direct comparison to a control group. Second, the sample size of the entire study was relatively small, thus all findings, especially those findings involving comparisons at the 1-year follow-up, should be considered with caution. Third, the participants enrolled came from an ‘all comers’ approach which intentionally led to a heterogenous sample of children with varying attention difficulties. While this approach capitalized on the practical nature of having this work occur at a school as an after-school program, it is distinct from other work that specifically excluded children whose objective attention functioning did not meet a given baseline criteria to ensure a homogenous study sample. This approach also warrants the need to replicate these findings with a cohort of children diagnosed with ADHD to interrogate the impact of the BBT platform in a homogenous population with known attention deficits. Fourth, it is important to note that only one of the reported correlations remained significant after correcting for multiple comparisons using a false discovery rate approach. While the observed correlations highlighted here provide an initial signal of benefits and preliminary insights into brain-behavior relationships they need to be replicated in future studies. Finally, it should be noted that the inter-trial coherence neural metric utilized here is distinct from related work that utilized midfrontal theta power^49^, as is the task that was performed while EEG recordings were taking place (here, the CPT task whereas the other studies utilized a perceptual discrimination task). As a point of reference, ITC reflects the consistency of activity at the trial level (thus, being more comparable to RTV or tau), whereas theta power describes that amplitude of neural engagement at stimulus onset. Our decision to focus on ITC was driven by the known issue of exacerbated performance variability when assessing pediatric populations, especially those with issues of inattention^86,87^, in conjunction with our decision to focus on RTV. It should be repeated that the interpretation of the findings presented should be taken with caution given that there may be other factors unaccounted for here that could have affected the outcomes of the study. These factors include, but are not limited to, unaccounted improvement in one’s personal health or outside activities that were unaccounted for.

Here we provide initial evidence for the utility of a novel digital intervention that combines physical and cognitive challenges towards the overarching goal of enhancing attention in children across a range of attention abilities. These findings contribute to the emerging field of digital therapeutics, demonstrating that these approaches may be beneficial to certain populations. Furthermore, the corroboration of the objective testing and parent reports with neural findings provide a specific target for future work to evaluate the neural mechanisms of observed changes. These approaches should not be evaluated in a vacuum; future work should evaluate the utility of each digital intervention with the perspective that individuals may benefit from different approaches. The present findings provide an initial vantage point regarding a non-tablet, motion capture digital therapeutic in children with issues of inattention.

## Methods

### Participants

Participating children had either: i) parental concerns of inattention, ii) a school or community-based diagnosis of ADHD, or iii) neither and were simply interested in participating. Children were asked to engage in 24 sessions of BBT training over 8 weeks (one child per training session, with each session being ~30 min of training, with 2 min breaks after each training run; see methods for more details), with a research assistant present for each session to monitor participation and provide support and feedback to the parents and children during training. This study was registered on the ISRCTN registry [ISRCTN59416198] as a retrospective trial, as the study was designed primarily to gauge the feasibility of using this intervention for a subsequent large-scale intervention trial, with reported effects on stipulated outcomes being a secondary goal. The study was approved by the Committee on Human Research at the University of California, San Francisco, who oversaw and approved all experimental protocols, with all methods performed in accordance with the relevant guidelines and regulations. The primary caregiver provided written consent on behalf of their child and the children provided informed assent, including for the taking and publication of photographs during study activities. With respect to Fig. 1, the authors affirm that participants provided informed consent for publication. To compensate for the time and effort of participation in this study, caregivers received $20 for each outcome assessment session, and $5 for each training session. With respect to participant characterization and screening, the Wechsler Intelligence Scale for Children Fifth Edition (WISC-V) was administered to all participants at the pre-training visit, with inclusion of children with Verbal Comprehension Index (VCI) ≥ 70. Children were excluded for prematurity (gestational age < 32 weeks), seizures requiring current medication management, or concern for Autism Spectrum Disorder as measured using the Social Communication Questionnaire (score > 15).

### BBT Intervention

BBT (Fig. 1a) integrates full body motion capture technology with cardiac and cognitive adaptive algorithms into a high-level (art, music, story) 3D video game targeting cognitive and physical fitness goals. As an overview, there are three BBT modules, with each targeting a different aspect of cognitive control: a visual search task for attention (with increasing distraction), a spatial span task for working memory, and a task-switching paradigm targeting cognitive flexibility abilities (see Fig. 1b).

The visual search module demands an active scan of the screen in search of a target, much like traditional visual search tasks. This module involves a constantly evolving amount of cued information as well as number of incongruent distracting elements, such that participants experience less cued information while experiencing more and more distracting elements as they advance. Participants are required to quickly identify the direction of a probe target that is facing at a right angle (up, down, left, right), and are aided by the presence of directional cue indicating in which location of the screen the target will appear amongst distracting elements. Responses are made by reaching their hands to indicate the direction of the probe, with the additional physical challenge of running in place if the target is up or down. Prior to each level, participants completed a thresholding session to determine the optimal starting point from both a cognitive and physical perspective. After completing their initial 7 training sessions, participants advance to Level 2 of this module which entailed facing a greater challenge: here participants encountered an increase in the number and salience of distracting elements, including the presence of congruent distractors, as based on their performance on the previous trial. After completing 14 training sessions, participants moved on to Level 3 of this module: here participants performed the same task as before, but now without the aid of a directional cue. Critically, participants only receive game points when they correctly perform a given trial faster than the predetermined, personalized threshold determined at the beginning of each level to optimize the attentional engagement.

The working memory module engages spatial working memory resources similar to the Corsi block task^88^, requiring individuals to memorize an additional stimulus following two consecutive correct responses, with two consecutive incorrect trials leading to one element being subtracted. Participants memorize the location of objects on screen followed by a 5-7 second delay period during which the participants perform a directed physical movement, with a correct response leading to a greater number of potential targets to be memorized on the next trial (and vice versa). Responses are made with both hands and feet by reaching/kicking targets, with additional physical challenges (making a “wood-chopping” motion) occurring during the delay period. Prior to each level, participants completed a thresholding session to determine the optimal starting point from both a cognitive and physical perspective. After completing 7 training sessions, participants are asked to also memorize and report the sequential order in which the targets originally appeared on the screen (Level 2), thus increasing the spatial working memory load. After 14 training sessions, participants perform a working memory/multiple object tracking task that requires memorizing and tracking the targets as they become invisible and move amongst a sea of moving objects (Level 3). As before, participants only receive game points when they correctly complete a working memory trial faster than a predetermined, personalized threshold so as to challenge the underlying cognitive working memory circuitry.

The task switching module challenges cognitive flexibility resources by requiring participants to rapidly switch their focus based on distinct rules, much like a traditional task-switching paradigm. Here a morphing algorithm is used to titrate the perceptual similarity of the target presented, such that a correct trial makes a subsequent exemplar morph more similar to the probe presented (and vice versa). Participants are presented with exemplar objects along with a target, and move their hands to the target object that is most similar to the exemplar presented. For example, when a greenish-blue target appears, participants decide whether the image is more green or more blue. The target changes its degree of likeness to each exemplar following each trial, with each correct response morphing the probe towards an indistinguishable 50/50 ratio of each exemplar (and vice versa). Prior to each level, participants completed a thresholding session to determine the optimal starting point from both a cognitive and physical perspective. After completing 7 training sessions, the presented probes now have features that integrate two rule bases (Level 2, e.g. both Color and Shape, so a Blue square), creating greater cognitive demands, similar to interference generated by a Stroop task. Finally, after 14 training sessions, participants perform the same task, but the exemplars now spawn in random locations across the screen, heightening the cognitive demands further by requiring visual search (Level 3). Once again, participants receive game points when they perform a trial as fast or faster than a predetermined, personalized threshold to pressure underlying goal-management circuitry.

BBT utilizes personalized and precise titrating of training through continuous, closed-loop adaptivity to drive game mechanics. This involves rapid performance-based assessment, feedback, reward, and modulated challenges to establish the optimal dynamic interactivity between the player and the game environment. This is a design approach used extensively in our work over the past 10 years^89–91^. Participants complete a thresholding procedure for each module and each level by completing three runs of each module, then the program computes the average performance on each, creating a personalized starting point for each training experience. Comparable to our previous work using cognitive measures alone^43,89,92^, here we integrate real-time adaptivity for both the cognitive and physical aspects of the gameplay.

For each cognitive task, difficulty scales on a trial-by-trial basis, with a correct trial performed within a threshold-determined response window leading to a response window shortened by 10msec, and an incorrect trial leading to a lengthening of the response window by 30 msec (thus, a 1 up/3 down staircase), in line with our previous work^10^. Each module also has a unique cognitive task, with one specific scaling aspect: i) the task switch module involves a morphing algorithm that titrates the perceptual similarity of the target presented, such that a correct trial makes a subsequent exemplar morph more similar to the probe presented (and vice versa), ii) the visual search module involves a constantly evolving amount of cued information as well as number of distracting elements, as based on our recent work^93^, such that participants experience less and less cued information while experiencing more and more distracting elements as they advance, and iii) the working memory module requires individuals to memorize an additional stimulus following two consecutive correct responses, with two consecutive incorrect trials leading to one element being subtracted. These cognitive adaptive algorithms are designed to assure participants remain at an ~80% rate of accuracy, a level that is not too easy or too hard, so that it is enjoyable.

On the physical side, difficulty is tied to the demands associated with the distance an individual must travel for a given response, and the amount of time allocated to complete this response. These movement-related aspects are directly responsive to whether heart rate is below/within/above a predetermined heart rate window (see below) to ensure a moderately intense workout that does not impede the ability to perform the cognitive task. For example, if one is playing the game below their assigned heart rate range, the software will automatically increase the distance that the participant has to move to respond with their hands/feet on each trial until their heart rate is within the specified range.

Participants respond with their hands and feet to the aforementioned cognitive tasks by engaging three physical domains (aerobic, balance, and flexibility). BBT uses an off-the-shelf Microsoft Xbox Kinect 2™ to collect movement-based kinematics in response to game-based challenges presented, and also involves the use of an Apple Watch™ to capture heart rate data which is incorporated in real time during game play to adjust the physical demands of the intervention. As an example, if a participant’s heart rate is below a pre-determined threshold on a given trial, the distance required to respond on the next trial is increased, causing the participant to move a greater amplitude (often lunging, jumping, or even sprinting to one’s side) and then quickly returning to a starting position in anticipation of the next trial. Similarly, if an individual was training at a heart rate greater than this pre-determined threshold, then the distances required to respond would decrease to lessen one’s movement amplitudes on a given trial. This algorithmic approach modulated a participant’s heart rate to try and ensure that their training was predominantly performed at their ideal physical training window. Participants receive physiological and cognitive feedback on a continual basis by incorporating real-time heart rate data and cognitive performance metrics into the software’s adaptive algorithms to titrate the demands and rewards of game play. This ensures that each participant is appropriately challenged and engaged during their training experience. Thus, the cognitive and physical tasks do not compete for cognitive resources—they work in concert towards a common task-based goal, overcoming a problem in previous studies where cognitive and physical fitness training were combined^94–102^.

For BBT, the appropriate heart rate window was pre-determined with respect to their personal fitness level using an age-appropriate VO_2_ max protocol. Using the equation from Mahar et al. (2018)^103^, we used performance on the PACER (see below) to estimate VO_2_ max to determine the initial physical difficulty level (and each subsequent level) of the BBT intervention:

$$VO2{\it{max}}\left( {mLO2/kg \ast {\it{min}}} \right) = 45.619 + \left( {PACER \ast 0.353} \right)-\left( {Age \ast 1.121} \right)$$

Participants completed four training sessions a week for a total of 6 weeks. The physical difficulty of the training increase from 50 to 60% of their VO_2_ max following the 1st week of training to 60–70% during the 2^nd^ week of training, with this reaching and staying at 70–80% of their VO_2_ max for weeks 3–8. This ramping structure allows participants to become accustomed to the game play and physical training aspects, with 70–80% being considered a moderately intense physical workout^104^. Furthermore, there is ample evidence that the positive effects of exercise on cognitive performance follow a U-shaped function^105–107^, with 75% VO_2_ max shown to be an optimal point for measures involving response time^106^. The adaptivity associated with heart rate allows for an increase (or decrease) in effort within a prescribed window as determined by the participants VO_2_ max calculation. Thus, an increase in HR reflects participants performing at a greater intensity within this prescribed heart rate window.

### Feasibility measures

To assess feasibility, we probed the following questions: i) how practical was setting up the BBT platform outside of the laboratory, ii) how many participants who began training withdrew from the study, and iii) what percentile of assigned training sessions were completed. To assess attention-related improvements, we collected several outcome measures: a parent report ADHD measure (Vanderbilt), objective performance-based laboratory measures (continuous performance task, CPT), and a neural measure (electroencephalography (EEG) recordings). Here we focused on measures of attention that have previously been used by our group to quantify improvements following digital interventions in children with issues of inattention^16,59,65^ (designated as ‘primary’ outcome measures). The use of these same measures also facilitates comparisons between the current study and prior work, thus providing important context to interpret improvements (Fig. 1c). All other outcome measures collected were designated ‘secondary’ measures of interest, including measures of physical fitness that were assessed due to the nature of the intervention. These designations are stipulated in our trial registration as well (ISRCTN registry [59416198]).

### Primary outcome measures

Our first primary outcome measures was the Vanderbilt Attention Deficit/Hyperactivity Disorder Parent Rating Scale (VADPRS), which utilizes information based on the *Diagnostic and Statistical Manual of Mental Disorders, 4th Ed*. (DSM-IV), was administered here to primarily assess changes in parent perception of inattention^16^, as in our other work^16,59,65^. This measure was collected from the participants’ primary caregiver prior to, immediately following the intervention, and at the 1-year follow-up. When completing this instrument, caregivers were instructed to “….please think about your child’s behaviors in the past 6 months, or since the last time this assessment was given.” Inattention concerns were assessed using the 1^st^ 9 questions on the Vanderbilt, where participants’ parents rated questions of inattention on a scale from 0 to 3, with 0 representing never having a concern, 1 having occasional concerns, 2 often having concerns, and 3 representing very often having concerns. Note that participants scoring a 2 or a 3 on at least 6 of these 9 questions in conjunction with a score of 4 or 5 on any of the performance questions (questions 48–55) are characterized as having the inattentive subtype of ADHD. Across a series of studies, this measure has shown to have strong reliability^108^ and validity^109,110^.

The next primary outcome measure was a measure of sustained attention that was derived from a well-validated continuous performance task (CPT), the Test of Variables of Attention (T.O.V.A.)^40,41^. This measure provides an index of sustained attention and impulsivity, and has shown to have strong reliability and validity for response time variability^41,111^. The experiment was programmed in Presentation (http://neurobs.com) and the stimuli were presented on a CRT monitor. For the present study, we adapted the task for use with EEG recordings, which requires many trials with a motoric response. In this task, participants maintain fixation on a central crosshairs and grey squares are shown on a black background at the top or bottom of the field of view. During the sustained condition, target stimuli were presented infrequently at the top of the screen as a 1:4 ratio of targets to nontargets and participants are instructed to only respond to these target stimuli. During the impulsivity condition, target stimuli were presented frequently at the top of the screen as a 4:1 ratio of targets to nontargets and participants are instructed to only respond to these target stimuli. Participants completed 2 blocks of 125 trials for each condition. Our primary variable of interest on this assessment was response time variability (RTV), as this particular metric has been shown to be sensitive to changes following a digital intervention^42,43,49,86^. This outcome measure was collected at baseline, training completion, and the 1-year follow-up. For completeness, we also describe other measures typically reported from this task, including response time (RT), d-Prime, and ex-gaussian tau (a metric related to RTV that quantifies attentional lapses by examining the distribution of long RTs^112,113^).

While participants performed the CPT task, EEG activity was recorded with Active Two head cap (Cortech-Solutions) with a BioSemi ActiveTwo 64-channel EEG acquisition system in conjunction with BioSemi ActiView software (Cortech-Solutions). Signals were amplified and digitized at 1024 Hz with a 16-bit resolution. Anti-aliasing filters were used and data were band-pass filtered between 0.01 and 100 Hz during data acquisition. Data was preprocessed using Analyzer software (Brain Vision, LLC), with blinks and eye-movement artifacts removed through an independent components analysis, as were epochs with excessive peak-to-peak deflections (± 100 mV). All EEG data underwent the same processing methodology as previously established by our lab^114,115^ to reveal specific neural signatures to guide subsequent interpretations^116–118^.

ITC is quantified by the unit “phase locking value” (PLV), which ranges between 0 and 1, with a value of 0 indicating that the phase synchrony is completely random, and a value of 1 indicating that the phase-locking is perfectly synchronized across trials. ITC is defined as: ITC(f,t) = $\frac{1}{n}\;\mathop {\sum}\nolimits_{k = 1}^n {|{{{\mathrm{Fk}}}}({{{\mathrm{f}}}},{{{\mathrm{t}}}})|}$ where t is a given time, f is the resolved frequency, n is the total number of trials, and Fk is equal to *e*^*i*φ^, which is the phase of the angle in polar coordinates. The ITC time series was created by resolving 4–40 Hz activity using a fast Fourier transform (FFT) in EEGLAB. After the time series was resolved, 50 ms bins following the onset of the stimuli were created from 0 to 600 ms. We selected a cluster of frontal electrodes (Fz, FPz, AF3, AF4, and AFz) based on previous literature that has used this same electrode cluster for similar analyses^16,42,43^. In each case, PLVs were controlled for individual state differences at each session by baseline correcting each individual’s PLVs using their −200 to 0 period (thus, relative PLV). Note that this outcome measure was also collected at the 1-year follow-up.

### Secondary outcome measures

Our first secondary outcome measure was the NeuroRacer multitasking assessment, where participants responded to a designated stimulus presented on a computer monitor (green circles) while ignoring all other color/shape combinations. Participants were exposed to 3 blocks of 36 target stimuli and 36 non-target stimuli, with each stimulus appearing on the screen for 400 ms and an inter-trial interval of 2000–3000 ms (with 500 ms jitter). A fixation cross was present on the screen at all times above the car and below the color/shape signs. Participants were instructed and reminded after each run to maintain focus on the fixation cross. The fixation cross provided performance feedback on each task: it turned green for 50 ms when the correct sign was selected within the time window or an irrelevant sign was ignored. When either of the aforementioned conditions were not met, it would turn red for 50 ms. For the NeuroRacer multitasking assessment, cognitive performance was evaluated using the signal detection metric of discriminability (d-Prime, or d’) in the form of a cost index. This index calculated the percentage change in d’ from when a participant performed a perceptual discrimination task by itself (‘single tasking’) versus when they performed this same task while concurrently performing a visuomotor tracking task (‘multitasking’). Thus, the equation for this index is as follows: (multitasking d’ – single-tasking d’/ single-tasking d’). Visuomotor tracking performance was measured by the amount of time that the participant was able to keep the car at the center of the road. Note that this behavioral outcome measure was also collected at the 1-year follow-up.

The next secondary outcome measure was a delayed recognition working memory task we have previously used in numerous studies^43,45,119,120^. Here we examined performance on the Ignore Distractor (ID) condition of this task, where participants were instructed to ignore a distracting stimulus while performing this task. More specifically, each trial began with the presentation of a face displayed for 800 ms, followed by a delay period (3 s), the presentation of a face stimulus as a distractor (800 ms), a second delay period (3 s), and the presentation of a face probe (1 s). The participants were instructed to make a match/nonmatch button press response at the probe as quickly as possible, without sacrificing accuracy. This was followed by a self-paced inter-trial interval (ITI). Our primary variables of interest on this assessment were accuracy and response time. The experiment was programmed in E-Prime (https://pstnet.com/products/e-prime/) and the stimuli were presented on a CRT monitor. Due to time restrictions, this measure was not collected at the 1-year follow-up.

The remaining secondary outcome measures were the physical fitness measures that include elements from the FitnessGram^52–58^, a field-test battery for youths used by the Presidential Youth Fitness Program that has established standards for ages 5–17 years. Participants performed a Curl-Up, 90 degree Push Up, Trunk Lift, and the PACER run to assess changes in fitness and strength. These measures were not collected at the 1-year follow-up.

Before the battery of measures was assessed, each participant performed a warm-up consisting of jumping jacks and running for 2 min prior to performing each of the fitness outcomes. Here we describe each of the measures and associated scoring: i) Curl-up: The subject lies on his/her back with knees bend at a 140-degree angle, with feet flat on the floor, legs slightly apart. The arms are kept straight along the body, palms facing down. The fingers are stretched out and the head is in contact with the floor. The fingertips are touching the tape that runs horizontally under the legs. During each curl-up, the subject lifts his/her head and upper chest toward the knees. The fingers should slide across the tape toward the ankles. Scoring on this task was calculated as the total number of curl-ups performed at a set pace (1 curl-up every 3 s) until a break was needed. ii) Push-up: The subject is positioned down on his/her hands and feet, facing down. The hands are placed under or slightly wider than the shoulder, fingers stretched out, legs slightly apart with toes tucked under. The back should be kept in a straight line from head to toe throughout the test. The subject lowers down until elbows are bent at a 90-degree angle and pushes up again. Scoring on this task was calculated as the total number of push-ups at a set pace (1 push-up every 3 s) until a break was needed. iii) Trunk lift: The subject lies down on the floor facing down with toes pointed and hands under the thighs. A marker is placed on the floor in line with the subject’s eyes. During the trunk lift, the subjects lift his/her upper body up to 12 inches while keeping straight spine and eyes focused on the marker. Scoring on this task was calculated via the distance from the floor to the subject’s chin; the maximum score on this test is 12 inches, anything over this distance is recorded as 12 inches. iv) Pacer run: The subject runs back and forth across a 20-meter distance at a specified pace that gets faster and faster. Participant heart rate was also captured during the PACER exercise period using an Apple Watch, with our primary measure of interest being maximal heart rate achieved during the PACER run exercise. The number of laps before a break was needed was recorded, with one point scored for each 20-meter distance covered.

Finally, we also collected a control measure in the form of a basic response time (BRT) task to help evidence that any improvements on the cognitive measures were specific to attention and working memory processes and not simply the result of a general increase in basic speed of processing.

In this task, participants respond to a target stimulus (40 trials) with a button press. Here we assessed response time and response time variability in line with our previous work^43^.

### Statistical analysis

Changes in cognitive control and survey measures were assessed with paired samples t-tests comparing: (1) pre- to post-training performance, (2) post-training to 1-year follow-up performance, and (3) pre-training to 1-year follow-up. This approach was taken (as opposed to a repeated measures ANOVA with all three timepoints) due to the small number of participants who completed all assessments at each timepoint (see Fig. 2). The goal of the comparisons of 1-year follow-up to both the post- and pre-training time points was to assess if those measures had changed 1-year later (post-training versus 1-year), as well as at the 1-year mark from when performance was initially evaluated at baseline (pre-training versus 1-year). To compare the present results versus the historical controls, we conducted repeated measures ANOVAs with a factor of session (pre, post) and between-group factor (BBT, TDC, SPD_IA_) on the primary behavioral measures of interest (Vanderbilt inattention score and CPT RTV), with follow-up independent sample t-tests for direct between group comparisons at each timepoint.

For the EEG ITC analyses, we conducted repeated measures ANOVAs with within-subjects factors of time window (0–600 ms via 50 ms bins) and session (pre and post), separately for each CPT task condition (impulsive and sustained), as this analysis allowed us to evidence training-related changes at specific time windows following stimulus onset as in our previous work^13^. Where a session by time window interaction was present, follow-up paired samples t-tests tests were conducted to identify which 50 ms time window (from 0 to 600 ms) showed a significant change between sessions. Statistical tests comparing post-training to 1-year follow-up for the EEG data were conducted using nonparametric Wilcoxon Signed Rank tests due to the small number of participants with available data (less than 10 in these cases).

The change in peak neural ITC (or change in ITC averaged across all time windows if no interaction was present) was entered into correlational analyses with our other primary metrics of interest. All correlations conducted reflect a Pearson product-moment correlation. While we present the results of all correlations here without any correction for multiple comparisons given the pilot nature of this work, we also mention which of these results would survive a false discovery rate (FDR) correction. Further, effect sizes for changes in our metrics of interest were calculated using Cohen’s d, and presented in Tables 1, 2. All statistical analyses were conducted using SPSS 22.0 (SPSS Inc.), with a *p*-value of 0.05 set as the threshold for significance.

### Reporting summary

Further information on research design is available in the Nature Research Reporting Summary linked to this article.

## Supplementary information

Supplemental Materials

REPORTING SUMMARY

## Data Availability

The datasets generated during and/or analyzed during the current study are available from the corresponding author upon reasonable request.

## Code availability

The code used in the analysis of the behavioral and EEG data was designed in Matlab R2022b (The MathWorks Inc.) and are available from the corresponding author on reasonable request.
