# Author Correction: Enhancing attention in children using an integrated cognitive-physical videogame: A pilot study

**DOI:** 10.1038/s41746-023-00826-7

**Published:** 2023-04-25

**Authors:** J. A. Anguera, M. A. Rowe, J. J. Volponi, M. Elkurdi, B. Jurigova, A. J. Simon, R. Anguera-Singla, C. L. Gallen, A. Gazzaley, E. J. Marco

**Affiliations:** 1 Neuroscape Center, Department of Neurology, University of California, San Francisco, USA; 2 Department of Psychiatry, University of California, San Francisco, USA; 3 Department of Neurodevelopmental Medicine, Cortica Healthcare, San Rafael, USA

**Keywords:** Attention, Cognitive control

Correction to: *npj Digital Medicine* 10.1038/s41746-023-00812-z, published online 12 April 2023

The original version of the Acknowledgements contained an error, in which inadvertently, one of the names of an individual acknowledged was incorrect, and acknowledgement was omitted. The correct version of the Acknowledgements is:

We would like to thank all the participating children and parents from Neil Cummings Elementary School for helping us with this study. We would also like to thank our funding for this project: the UCSF RAP Academic Senate as well as our Neuroscape donors (Neuroscape Network), who provided us the freedom to undertake and complete this project. We would also like to thank Ms. Patty Elliott (principal at Neil Cummings Elementary School) for allowing us to work with her on this project, Ms. Vuolo (occupational therapist at Neil Cummings) for helping oversee our training efforts, R. Campusano for assisting with EEG set up, Delilah Mittermaier for hands on pediBBT data collection, and C. Thompson for help thinking through the most practical physical fitness outcome measures for this study.

This replaces the previous incorrect version of the Acknowledgements:

We would like to thank all the participating children and parents from Neil Cummings Elementary School for helping us with this study. We would also like to thank our funding for this project: the UCSF RAP Academic Senate as well as our Neuroscape donors (Neuroscape Network), who provided us the freedom to undertake and complete this project. We would also like to thank Ms. Patty Smith (principal at Neil Cummings Elementary School) for allowing us to work with her on this project, Ms. Vuolo (occupational therapist at Neil Cummings) for helping oversee our training efforts, R. Campusano for assisting with EEG set up, and C. Thompson for help thinking through the most practical physical fitness outcome measures for this study.

This has been corrected in both the PDF and HTML versions of the Article.

