# Supplementary material for: Enhancing attention in children using an integrated cognitive-physical videogame: A pilot study
Source: NPJ Digit Med. 2023 Apr 12;6:65. doi: 10.1038/s41746-023-00812-z (PMC10097690; doi:10.1038/s41746-023-00812-z)
Supplement: Supplementary file 1 — Supplemental Materials [file 41746_2023_812_MOESM1_ESM.pdf]

Supplementary Materials

Supplementary Table 1. Participant demographics at enrollment

	**Mean (SD)**	**N (number male)**
**Age**	9.24 (1.57)	22 (16)
**WISC-V VCI**	107.36 (10.44)	22 (16)
**WISC-V VSI**	104.25 (17.60)	20 (14)
**WISC-V FRI**	103.75 (10.96)	20 (14)
**WISC-V WMI**	101.38 (11.69)	21 (16)
**WISC-V PSI**	98.16 (9.19)	19 (14)
**WISC-V FSIQ**	104.59 (11.16)	22 (16)
**SSP Total**	154.59 (16.09)	22 (16)
**SCQ Total**	4.45 (3.80)	22 (16)
**Satisfaction Score**	81.79 (18.70)	19 (13)

Supplementary Table 2: Correlation Matrix assessing the change over time on each Primary Outcome Measure.

	Vanderbilt	CPT Impulsive RTV	CPT Sustained RTV	CPT Impulsive ITC	CPT Sustained ITC
**Vanderbilt**	-	-	-	-	-
**CPT Impulsive RTV**	r= 0.156<br>p= .524<br>n= 19	-	-	-	-
**CPT Sustained RTV**	r= 0.282<br>p= .242<br>n= 19	r= .056<br>p= .819<br>n= 19	-	-	-
**CPT Impulsive ITC**	**r= 0.742**<br>**p= .004***<br>**n= 13**	r= .378<br>p= .252<br>n= 11	r= .232<br>p= .493<br>n= 11	-	-
**CPT Sustained ITC**	r= 0.058<br>p= .844<br>n= 14	**r= .580**<br>**p= .048**<br>**n= 12**	r= .204<br>p= .525<br>n= 12	r= .028<br>p= .927<br>n= 13	-

*** => p-value survived False Discovery Rate correction (p= .040)**

Does your child struggle with attention?

Can a smart video game help?

UCSF researchers are partnering with Neil Cummins to study whether training the mind and body, with video game technology, can help attention and memory.

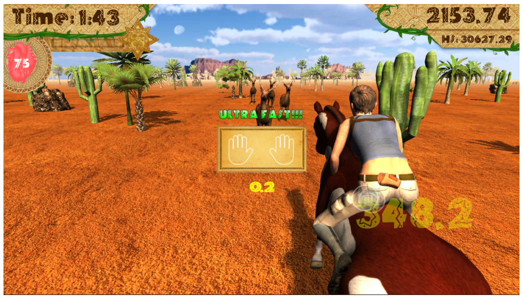

To participate, kids must be:

- 7-12 years of age
- Able to participate in brain imaging and cognitive testing at UCSF (in San Francisco) before and after training
- Willing to play a fun motion capture "video game" at Neil Cummins 3 times a week for 8 weeks

For more information, contact our study coordinator:

Molly.Gerdes@ucsf.edu or (415) 640-2680

NEIL CUMMINS

UCSF

Supplementary Figure 1. **Recruitment flyer/email for the study**

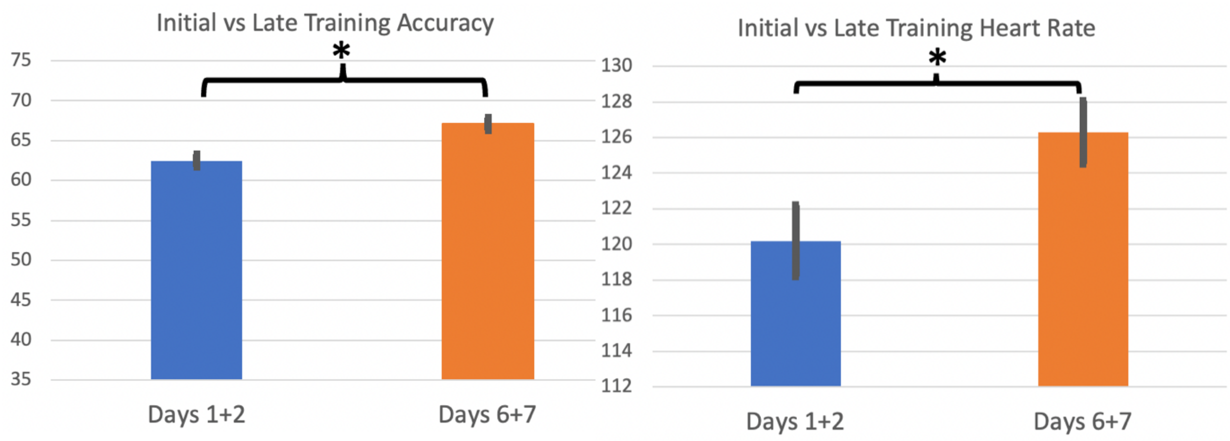

Supplementary Figure 2. Change in BBT training accuracy and heart rate when collapsed across modules (3) and session (7 sessions per module). Here we observed a difference between the initial training sessions (the average performance over the 1st two days of training versus later training sessions (the average performance over the final two days of training), after collapsing across modules and levels in terms of accuracy ($t_{(21)}$ = -7.3, $p < 0.001$) and heart rate ($t_{(21)}$ = -3.5, $p = 0.002$).
