# Supplementary material for: Enhancing attention in children using an integrated cognitive-physical videogame: A pilot study
Source: NPJ Digit Med. 2023 Apr 12;6:65. doi: 10.1038/s41746-023-00812-z (PMC10097690; doi:10.1038/s41746-023-00812-z)
Supplement: Supplementary file 2 — REPORTING SUMMARY [file 41746_2023_812_MOESM2_ESM.pdf]

nature portfolio

Corresponding author(s): Anguera, Joaquin A.

Last updated by author(s): Feb 11, 2023

Reporting Summary

Nature Portfolio wishes to improve the reproducibility of the work that we publish. This form provides structure for consistency and transparency in reporting. For further information on Nature Portfolio policies, see our Editorial Policies and the Editorial Policy Checklist.

Statistics

For all statistical analyses, confirm that the following items are present in the figure legend, table legend, main text, or Methods section.

n/a	Confirmed	
☐	☒	The exact sample size (*n*) for each experimental group/condition, given as a discrete number and unit of measurement
☐	☒	A statement on whether measurements were taken from distinct samples or whether the same sample was measured repeatedly
☐	☒	The statistical test(s) used AND whether they are one- or two-sided<br>*Only common tests should be described solely by name; describe more complex techniques in the Methods section.*
☒	☐	A description of all covariates tested
☐	☒	A description of any assumptions or corrections, such as tests of normality and adjustment for multiple comparisons
☐	☒	A full description of the statistical parameters including central tendency (e.g. means) or other basic estimates (e.g. regression coefficient) AND variation (e.g. standard deviation) or associated estimates of uncertainty (e.g. confidence intervals)
☐	☒	For null hypothesis testing, the test statistic (e.g. *F*, *t*, *r*) with confidence intervals, effect sizes, degrees of freedom and *P* value noted<br>*Give P values as exact values whenever suitable.*
☒	☐	For Bayesian analysis, information on the choice of priors and Markov chain Monte Carlo settings
☒	☐	For hierarchical and complex designs, identification of the appropriate level for tests and full reporting of outcomes
☐	☒	Estimates of effect sizes (e.g. Cohen's *d*, Pearson's *r*), indicating how they were calculated

Our web collection on statistics for biologists contains articles on many of the points above.

Software and code

Policy information about availability of computer code

Data collection	Eprime V2.0, Matlab Psychophysics-3 Toolbox; Presentation (http:// neurobs.com); Biosemi's ActiView EEG recording software
Data analysis	Matlab + IBM SPSS v20

For manuscripts utilizing custom algorithms or software that are central to the research but not yet described in published literature, software must be made available to editors and reviewers. We strongly encourage code deposition in a community repository (e.g. GitHub). See the Nature Portfolio guidelines for submitting code & software for further information.

Data

Policy information about availability of data

All manuscripts must include a data availability statement. This statement should provide the following information, where applicable:

- Accession codes, unique identifiers, or web links for publicly available datasets
- A description of any restrictions on data availability
- For clinical datasets or third party data, please ensure that the statement adheres to our policy

The data that support the findings of this study are available from the corresponding author upon reasonable request.

Human research participants

Policy information about studies involving human research participants and Sex and Gender in Research.

Reporting on sex and gender	Findings do not apply to only one sex or gender. We were agnostic to sex and gender when designing this study, with gender being self-reported by each participant's parent(s)/guardian(s). With respect to gender, a Chi-square test (Chi-square value = 2.98, p= .23) revealed no difference in gender (that is, the number of participants by gender) between the current and 2 historical data sets (from Anguera et al. (2017; PLOS ONE)). Thus we chose to not use gender as a covariate. Note that we used the same approach in our Anguera et al. (2017; PLOS ONE) publication which led us to not use gender as a covariate there either.
Population characteristics	A total of 22 children (6 female) ages 7 to 12 years (mean age= 9.24, SD= 1.57) completed the intervention, with 16 contributing data to the 1-year follow-up assessment
Recruitment	Children were recruited from Neil Cummins Elementary School in Corte Madera California, if they were between the ages of 7 and 12 years through school postings, newsletters, and word of mouth
Ethics oversight	The study was approved by the Committee on Human Research at the University of California, San Francisco

Note that full information on the approval of the study protocol must also be provided in the manuscript.

Field-specific reporting

Please select the one below that is the best fit for your research. If you are not sure, read the appropriate sections before making your selection.

☒ Life sciences ☐ Behavioural & social sciences ☐ Ecological, evolutionary & environmental sciences

For a reference copy of the document with all sections, see nature.com/documents/nr-reporting-summary-flat.pdf

Life sciences study design

All studies must disclose on these points even when the disclosure is negative.

Sample size	Here we estimated the sample size by looking at our previous studies (Anguera et al., 2017; Gallen et al., 2021), and looking to enroll a comparable number of participants given the use of the same outcome measures in each case.
Data exclusions	In Figure 2 (the CONSORT diagram), we illustrate the number of participants at different stages of the study, from consent to enrollment and 1-year follow-up. The embedded table reflects the number of participants with datasets for each of the primary measures of interest at each time point, as well as the number of individuals with data at each paired timepoint. For the CPT task, missing data was due to participants not being able to stay to the end of their testing session due to other outside obligations those days. For the EEG recordings, missing data points reflect a hardware error where photodiodes used to time lock the onset of targets were not functioning properly, preventing the analysis of event-related activity.
Replication	Given the longitudinal nature of the study and human subjects population, no attempts were made at replication.
Randomization	This was a single-arm open label pilot study, thus randomization was not employed.
Blinding	This was a single-arm open label pilot study, thus blinding was not employed.

Reporting for specific materials, systems and methods

We require information from authors about some types of materials, experimental systems and methods used in many studies. Here, indicate whether each material, system or method listed is relevant to your study. If you are not sure if a list item applies to your research, read the appropriate section before selecting a response.

Materials & experimental systems

n/a	Involved in the study
☒	☐ Antibodies
☒	☐ Eukaryotic cell lines
☒	☐ Palaeontology and archaeology
☒	☐ Animals and other organisms
☐	☒ Clinical data
☒	☐ Dual use research of concern

Methods

n/a	Involved in the study
☒	☐ ChIP-seq
☒	☐ Flow cytometry
☒	☐ MRI-based neuroimaging

Clinical data

Policy information about clinical studies

All manuscripts should comply with the ICMJE guidelines for publication of clinical research and a completed CONSORT checklist must be included with all submissions.

Clinical trial registration

ISRCTN 59416198

Study protocol

https://www.isrctn.com/editorial/retrieveFile/a21151cb-49a6-4624-a8d6-49da20d418cb/39914

Data collection

Recruitment and data collection start date = 20/01/2018

Recruitment and data collection end date = 20/02/2020

There were 3 primary locations:

Intervention Site: Neil Cumming Elementary School
58 Mohawk Ave
Corte Madera
94925
United States of America

Outcome Assessment Site: University of California, San Francisco
675 Nelson Rising Lane
San Francisco
94080
United States of America

Outcome Assessment Site: Cortica Marin
4000 Civic Center Drive, STE 100
San Rafael
94903
United States of America

Outcomes

Primary outcome measure (as defined in our trial registration)
1. Parent report of inattention using the Vanderbilt
2. Objective measure of attention (RTV) using the Continuous Performance Task (CPT)
3. Objective measure of attention (ITC) using EEG during the Continuous Performance Task (CPT)

Secondary outcome measures (as defined in our trial registration)
1. Objective measure of Multitasking using the NeuroRacer Multitasking Assessment
2. Objective measure of Working Memory using the delayed recognition working memory task (AID)
3. Objective measure of Basic Response Time (BRT) using the BRT task
4. Surveys of physical fitness
5. Surveys of general health
